# Medicinal Plants of the Maasai of Kenya: A Review

**DOI:** 10.3390/plants9010044

**Published:** 2019-12-27

**Authors:** Jedidah Nankaya, Nathan Gichuki, Catherine Lukhoba, Henrik Balslev

**Affiliations:** 1 School of Biological Sciences, University of Nairobi, P.O. Box 30197-00100 Nairobi, Kenya; nankaya@mmarau.ac.ke (J.N.); ngichuki@uonbi.ac.ke (N.G.); clukhoba@uonbi.ac.ke (C.L.); 2 School of Natural Resources and Animal Sciences, Maasai Mara University, P.O Box 861-20500 Narok, Kenya; 3 Department of Bioscience—Ecoinformatics and Biodiversity, Aarhus University, Build. 1540, Ny Munkegade 116, DK-8000 Aarhus C, Denmark

**Keywords:** ethnobotany, traditional medicine, traditional knowledge, medicinal use category, traditional practices, traditional use patterns

## Abstract

The use of medicinal plants for treatment of humans and animals is entrenched in the Maasai culture and traditional knowledge related to it is passed on from one generation to the next. A handful of researchers have in the past decades documented this knowledge. No single study has documented medicinal plant uses of the Maasai community as a whole. This review provides a consolidated database of the diversity and uses of medicinal plants among the Maasai in Kenya. The study will help conserve traditional medicinal plant knowledge that is valuable for the development of modern medicine. Relevant information on medicinal plants used by the Maasai of Kenya was extracted from journals, books, M.Sc., and Ph.D. dissertations. We found evidence of 289 plant species used by the Maasai of Kenya in traditional medicine. Most species were used to treat health conditions in the categories *gastrointestinal* and *respiratory system disorders*. The most used families were Leguminosae, Asteraceae, Malvaceae, Euphorbiaceae, and Lamiaceae. Medicines were commonly prepared as a decoction and administered through oral ingestion, with roots reported to be the preferred plant part for medication. The Maasai preference for roots compared to other plant parts may be unsustainable and could threaten species availability in the future.

## 1. Introduction

All humans depend on plants for food, medicine, firewood, and more [1]. The use of medicinal plants has played an important role in traditional cultures in alleviating human suffering [2]. Nowhere is this more prominent than on the African continent where 80% of the population use traditional medicine to treat sickness and for maintaining good health [3]. Kenya is not an exception; although there exist other forms of heath care providers such as the government hospitals, clinics, private and faith-based health facilities, the rural population relies on traditional medicine in innumerable ways for their health care needs [4]. The preference often given to a traditional pharmaceutical system is attributed to the efficacy of traditional medicinal care in the treatment of some diseases, ease of availability, and affordability compared to western medicine, and in addition traditional uses are culturally more acceptable [2,5,6]. This all shows that local medicinal knowledge can contribute to the alleviation of a broad variety of health care problems in both rural and urban populations who complement modern treatment with traditional therapies.

People around the world have different cultures and lifestyle which influence medicinal use patterns and practices. The parts of the plants used and the preparation methods could also be influenced by species availability and traditional knowledge of use. To understand diversity of medicinal plants, all these factors should be considered.

Traditional knowledge of medicinal plants held by different communities has provided baseline data for selection of species for screening of active compounds in search of new drugs [7,8]. Nevertheless, the use of plants for traditional medication has a number of shortcomings including efficacy concerns, uncertainty concerning dosage, diagnosis challenges, and changing plant species availability [2].

**Figure 1:**
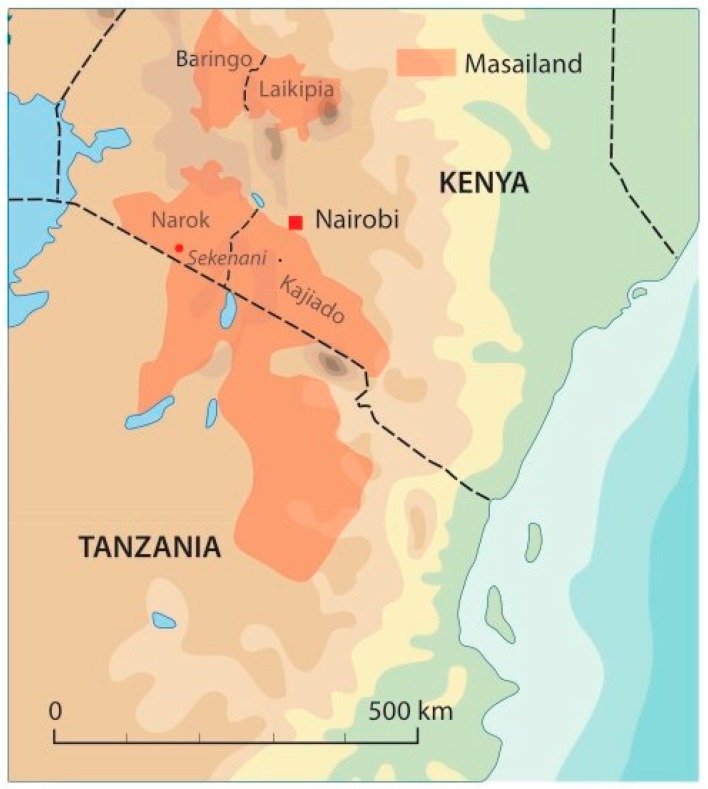
The extension of the Maasai territory and their counties in Kenya. Original artwork by Flemming Nørgaard, Aarhus University.

The Maasai live as pastoralists in Kenya and Tanzania, and their population is estimated to be about 1 million. The Maasai keep cattle as their main source of wealth and food [9]. In Kenya, the Maasai live mainly in the Kajiado, Narok, Baringo, and Laikipia counties and they speak the Maa language (Figure 1).

In the Maasai community, traditional use of plants for curative purposes is a cultural practice that has been inherited from their ancestors over many generations [10]. For example, young Maasai warriors, the Morans, carry out traditional ceremonies that involve the slaughter of bulls deep in the forest where they gather medicinal plants to prepare a traditional soup for general body health. The Maasai community is known to possess a rich traditional knowledge on the use of plants for medication dating back probably for centuries, but first documented only in the beginning of the last century [11,12]. They spend most of their time herding in the savannas and forests searching for fresh pastures and water for their livestock. As a result, they have a good understanding—acquired over time—of their local surroundings and natural resources. Of most importance are the plant species in their local environment which provide medicine, construction materials, firewood, and fodder for their cattle.

Due to strong cultural beliefs, an expensive government and private healthcare system, and long distances to available facilities, a majority of the Maasai, especially those in rural areas, prefer to use medicinal plants for their health care needs and for those of their livestock. Gastrointestinal disorders, parasitic infections, tuberculosis, brucellosis, sexually transmitted diseases, and wounds are the main diseases that affects the Maasai [13]. Some of the diseases could be as a result of the Maasai’s close contact with cattle. For example, parasites such as worms and brucellosis could be a result of consumption of uncooked or semi-cooked milk, meat, and raw blood.

Nevertheless, the Maasai’s traditional way of life is rapidly changing due to the introduction of western education, increasing access to government healthcare facilities, climate change, and urbanization, among other factors, which all lead to the loss of this important knowledge and of the associated plant resource base [14]. A recent study conducted in Sekenani, Maasai Mara reported loss of plant knowledge and a decline in the use of medicinal plants in the area [15]. Whereas many of these changes are to the better for the Maasai, the loss of traditional knowledge may have undesired lateral effects that limit the well-being of the community because it may cause reduced cultural identity and loss of, for instance, free and easily accessible medicines from their environment.

The available research on medicinal plants of the Maasai of Kenya is highly fragmented, encompassing single villages in different geographical areas of the Maasai land. A consolidated account and analysis of the uses and use pattern of medicinal plants among the Maasai community as a whole is not available. Therefore, there is need for a comprehensive analysis and compilation of the available scientific literature concerning medicinal plants practices and use patterns of the Maasai people in Kenya. It is also important to understand whether medicinal plant knowledge is shared in different areas occupied by the Maasai in Kenya.

Such a compilation will aid in medicinal plant conservation, and could be used as a basis for subsequent studies on the pharmacology and phytochemistry of the highlighted plants for future development of drugs.

In our analysis, we describe and evaluate the diversity and use patterns of medicinal plants in the Maasai community of Kenya, specifically, we ask the following: (1) How many medicinal plant species have been documented for the Maasai community in Kenya, which plant species are most used for medicinal purposes, and which plant families do they belong to? (2) What are the ethnopharmacological uses of medicinal plants with high use reports, and which medicinal use categories do they belong to? (3) What parts of the plants are used, and how are the plant-derived medicines prepared and administered? (4) How homogenous is the use of medicinal plants among the Maasai of Kenya?

## 2. Methods

### 2.1. Data Collection

Data on medicinal plants used by the Maasai community were gathered from journals, books, published M.Sc. and Ph.D. theses, and in different electronic databases including science direct, Medline, Google scholar, Web of Science, AJOL, in local Kenyan university websites, the University of Nairobi, and Aarhus University libraries. We searched for relevant information using the key words “medicinal”, “plants of the Maasai”, “Maasai of Kenya”, “ethnobotany of the Maasai”, “traditional medicinal plants of the Maasai”, “indigenous knowledge of the Maasai”, and “local knowledge of medicinal plants of the Maasai”. In total we identified 15 articles, two theses (M.Sc. and Ph.D.) and two books, all published between 1994 and 2018 [9,13,14,16,17,18,19,20,21,22,23,24,25,26,27,28,29,30,31].

Before including the data in the database, it was traced to the original sources. We consolidated a database, organized in Excel, with columns for the scientific name of the plants, the reference from which data was obtained, counties where original research was conducted, villages and study sites when available, original use reports, parts of the plants used, preparation methods, route of administration, and classification of the condition treated.

We excluded use reports for plants that were not referred to by their scientific names. Plants that were referred to by their genus names only were also excluded, as it was not possible to know the exact species being referred to.

In this analysis, “use report” refers to the local uses of medicinal plants as reported in the primary reference [32]. So, a “use report” is the reference to a particular plant species for a particular medicinal treatment in a particular publication. The number of use reports was used to estimate the importance of the species in each category of illness. In cases where species had more than one use mentioned in the reference, each use was recorded as a separate use report. To avoid duplication, the cited literature was carefully checked to ensure that the mention of a species for a particular use was original and not referring to a previous publication. When we encountered use reports from secondary sources they were traced to their original primary sources.

Scientific names from primary sources were verified and updated to current accepted names according to *The Plant List* [33]. Medicinal species were assigned to family following the Angiosperm Phylogeny Website [34].

In the cases where the names given in the primary references were listed as a synonym in the plant list, we databased them under their accepted names.

We found 177 use reports under synonyms and 1200 misspelled names in the primary sources which were then updated to the current accepted scientific name.

Some reported more than one plant part, for example, stem and roots, as ingredient in medicine. In such cases, we recorded the entry as multiple parts. When the used plant part was not provided in the primary sources, we recorded it as unspecified. When the whole plant was used, we recorded it as whole plant.

### 2.2. Commonly Used Medicinal Plants and Homogeneity of Use

To determine common species used and homogeneity of use in different localities, we calculated the number of use reports per species and Informant Consensus Factor (ICF) [35], respectively.

(1) $$\mathrm{ICF}=\frac{\mathrm{Nur}-\mathrm{Nt}}{\mathrm{Nur}-1}.$$

where Nur is the number of use reports in a particular category and Nt is the number of species that were used as medicine in a particular category. ICF values close to one indicates that there is homogeneity in the use of medicinal plants between the informants (i.e., the Maasai from different localities use the same medicinal species for the same disorder), while ICF values close to 0 suggests that there is little or no exchange of knowledge between informants (i.e., knowledge is not shared) [36,37]. In this analysis, we tested whether knowledge is shared between the localities inhabited by the Maasai people.

## 3. Results

### 3.1. Diversity of Medicinal Plants

**Figure 2:**
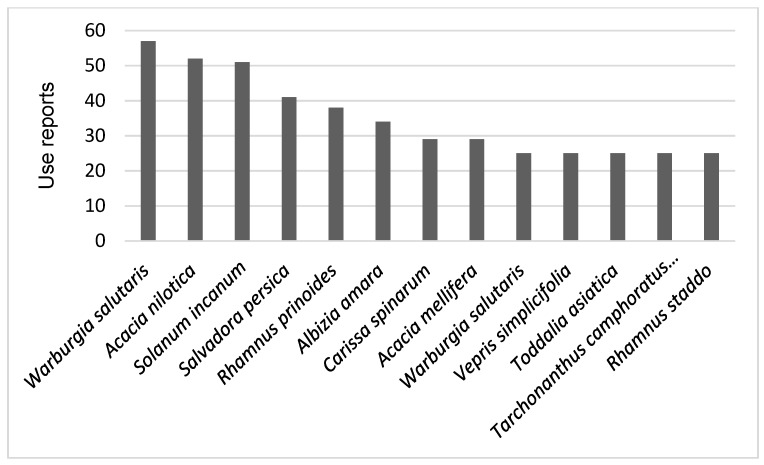
Thirteen medicinal species with 25 or more use reports cited in 19 references concerning Maasai medicinal plants in Kenya and reviewed in this study.

We found information about 289 medicinal plant species used by the Kenya Maasai which together had 1844 use reports (Supplementary material). Thirteen medicinal species had > 25 use reports (Figure 2). Leguminosae was the family with most species used as medicine by the Maasai, followed by Asteraceae, Malvaceae, Lamiaceae, and Euphorbiaceae. Noticeably, 22 families (31%) were represented by only one species with medicinal uses in our data (Table 1).

### 3.2. Use Categories with High Numbers of Use Reports Per Species

The 1844 use reports were classified into 26 health disorder categories following the International Classification of Primary Care classification system (ICPC) [38]. Most use records were in the categories Gastrointestinal disorders (504 use reports; 27%) *and* Respiratory system disorders (252 use reports; 14%) (Table 2). Nine disorders had more than five use reports per species (Table 3).

### 3.3. Plant Parts Used, Preparation and Route of Administration

**Figure 3:**
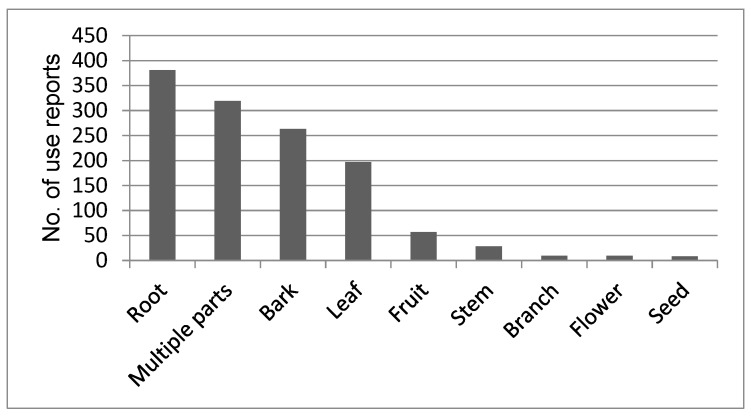
Plant parts used for preparing medicine among the Maasai of Kenya as cited in 19 references.

About two thirds of the primary sources analyzed mentioned which part of the plants that was used, but only 342 (19%) of the use reports mentioned how the plants were prepared before being used and their route of administration. Roots were the most frequently used plant part, followed by “multiple parts” (Figure 3). Decoction was the most common preparation method with 230 use reports, followed by pounding (76) and burning (29). The Maasai make a soup prepared by boiling pieces of meat in water with medicinal plants added for body health and strength. Occasionally, milk, honey, or tea is added to reduce the bitter taste. The least used methods were eating without any preparation (4) and soaking (3). Oral administration was the most common route of applying medicine with 25% of use reports followed by smearing, inhaling, and poultice. For the Maasai, the boiled content is ingested orally and the taste adjusted by adding milk or tea to the medicine. Medicinal plants were also applied directly to wounds or on the skin by smearing or rubbing the squeezed medicinal plant extract directly to the affected areas. Body steaming and bathing, dropping the medicine in the nose, eye, or ear were also used. However, 68% of the total use reports did not indicate the route of administration.

### 3.4. Homogeneity of Use

The use report data originated from two groups of the Maasai occupying Narok and Kajiado counties. Metabolic disorders, social problems, gastrointestinal, respiratory, and musculoskeletal disorders had the highest ICF values. The rest of the categories had low ICF (Table 2). The categories ear, circulatory, endocrine, psychological, immune system, and culture- bound disorders had a strikingly low ICF of 0.0 likely because of the complexity of diagnosing health conditions in these categories, which therefore remain unreported.

## 4. Discussion

### 4.1. Diversity of Medicinal Plants

**Figure 4:**
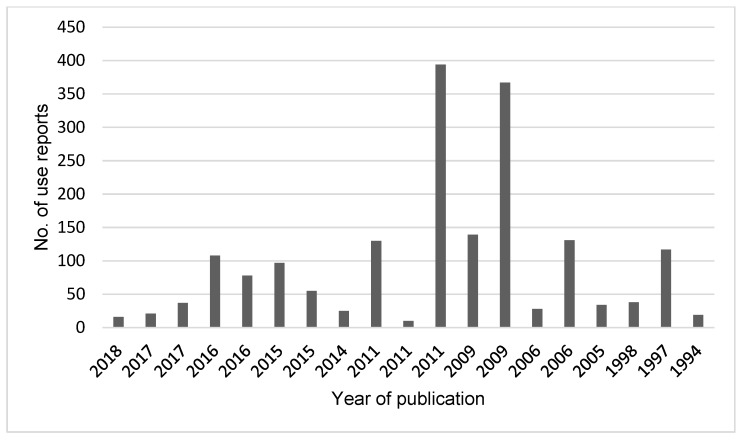
Year of publication and number of use reports for all the 19 references.

The use of plant-based medicine among the Maasai is still an important component of their culture. This is demonstrated by the high number of use reports in the primary references (Figure 4). It is a solid indication of the diversity of medicinal plants used by this community. The high reliance of medicinal plants may be due to strong cultural beliefs, high cost of other kinds of health care in Kenya, inaccessibility of governmental health facilities, and the quick and free accessibility of medicinal plants. The same reasons have been reported among the Samburu of Kenya [39] and in rural communities in southern Ecuador [40].

From the 1844 use reports gathered from the 19 references included in the analysis, 289 species were identified as belonging to 70 families. It was noted that all the data accrued from documented research were conducted in Narok and Kajiado counties suggesting that medicinal plant uses in other places such as Laikipia and Baringo counties have not yet been documented, published or the data is not available online or in hard print.

Out of the 289 species, 51 (18%) had ten or more medicinal use reports and 76 species had a single use report. Medicinal species with high numbers of use reports might be much more effective in treatments or could be more easily accessible compared to those with few use reports. On the contrary, those with single use reports could still be effective in traditional treatments but may not be easily available in the surrounding environments.

Leguminosae, Asteraceae, Malvaceae, Lamiaceae, and Euphorbiaceae were the plant families with most species used for medicinal purposes. Our findings partly agree with research conducted in Tanzania which reported Leguminosae, Lamiaceae, and Asteraceae to be dominant families [41]. The common use of species in the family Leguminosae, Asteraceae, Malvaceae, Lamiaceae, and Euphorbiaceae could be due to their high species diversity. Species with high diversity are more available and visible to people hence are likely to be preferred for use resulting in high numbers of use reports [42]. In Kenya, Leguminosae is the largest family with 576 species, Asteraceae (403), Malvaceae (219), Lamiaceae (206), and Euphorbiaceae (219) and were listed among the 13 families with the largest number of species [43]. Species in the family Asteraceae and Lamiaceae are herbaceous and inhabit disturbed areas as weeds, and are hence readily available [44].

### 4.2. Use Categories with High Use Reports Per Species

The most common category of health conditions treated with medicinal plants by the Maasai of Kenya was gastrointestinal disorders. More than half of all recorded medicinal species (59%) were used to treat gastrointestinal disorders which contributed 27% of the total use reports. The category had a high informant consensus factor of 0.66 indicating that the knowledge of use of medicinal plants is largely shared among the Maasai of Kenya occupying different localities. Kajiado and Narok counties are classified as arid and semi-arid counties with little or no access to clean water. Gastrointestinal disorders such as diarrhea are therefore common. Diarrhea is a common disease in Africa and is reported to cause death in children. Similar results were recorded in a study on medicinal uses of African palms [45].

Respiratory disorders were the second most common disorder treated with medicinal plants and had a high informant consensus value (0.64) and about 14% of use reports were classified into this category. In Ethiopia, 16% of the reported medicinal plants species were used to treat respiratory disorders [46].

Muscular-system disorders were the third common disorders that were treated with medicinal plants by the Maasai. This could be a result of the Maasai way of life which entails trekking long distances in search of pasture and water for their livestock. The long walks in the forest and savannas may result in body discomfort such as body pain, muscle aches, and rheumatism.

Malaria was the fourth disorder treated with medicinal plants. Malaria is caused by parasitic protozoa and is reported to cause over a million deaths in Sub-Saharan Africa [47]. We expected Malaria to be common among the Maasai due to their pastoral way of life; they live in areas with tall grass and near water sources which act as mosquitoes’ breeding sites, and hence are susceptible to Malaria.

Urological, gynecological, and skin disorders had only two species with more than five use reports and a low informant consensus factor of 0.47, 0.46, and 0.33 respectively. Neurological and toothache disorders also showed lower intracultural consensus due to few use reports. This suggests that the knowledge of medicinal plants in the five categories is not shared across geographical locations or within the Maasai groups. The difference in the use of medicinal plants in the studied counties could be a result of the ease of availability and ecological factors [48,49]. Similar findings have been reported from northwestern South America where knowledge on medicinal uses of palms was not shared among individuals of a cultural group [50].

### 4.3. Plant Parts Used, Their Preparation and Administration

The Maasai used many different plant parts for preparing medicine. We found reports for the use of eight different plant parts (Figure 2) but many reports did not specify the part used. In our data, roots were the most cited plant part used which agrees with studies conducted elsewhere in Africa [51,52]. The preference for using the roots for medication could be as a result of higher concentration of active ingredients than in other parts of the plant [32,53]. This contrasts with various other studies conducted in different parts of the Africa [54,55,56,57] that reported leaves as the most frequently used plant part for medication. In those studies, the preference for leaves was said to be for their abundance and because leaves contain high concentrations of compounds with various medicinal properties. Compared to leaves, however, roots have the ability to maintain bioactive compounds for a long time after harvesting [32]. The same argument could be true for the Maasai who prefer roots as they can be stored and used later or during harsh environmental conditions such as long droughts when fresh material is not available. Harvesting of roots and whole plant has the potential to damage the plant and could be unsustainable for some species [37,58] and more unsustainable than harvest of leaves and fruits which are constantly produced and can be harvested without causing irreversible damage to the plant [37].

Different methods of preparation of medicinal plants are used in various parts of the world. For the Maasai people in Kenya, we found that decoction was the most frequently used method of preparation. This is consistent with studies conducted elsewhere that reported decoction to be the most commonly used method for preparation [41,57,59]. Decoction might be the preferred method because of the ease of preparation and the taste of the boiled medicine could easily be adjusted by adding a solvent of choice, mostly water, honey, or milk.

The Maasai’s common route of administering the plant derived medicine was oral by drinking, which is the same as found in many other studies [60,61,62]. Even though we present an overview of ways in which medicinal plants were administered, there was missing information for over half of the use reports, which is a common data gap in many ethnomedicinal studies that was also noted by Farnsworth almost 30 years ago [63].

### 4.4. Homogeneity of Use

Metabolic disorders, social problems, gastrointestinal, respiratory, and musculoskeletal disorders had the highest ICF values, indicating that the plant species used for treatment in these categories were shared by informants [36,37]. The other 21 categories had lower ICFs. This suggests that the knowledge of medicinal plants is not necessarily shared across geographical locations or within groups. The difference in the use of medicinal plants in the studied counties could be a result of the ease of availability and ecological factors [48,49]. Our results agreed with a recent study conducted in Thailand [32], that found that each studied village had its own unique ethnomedicinal knowledge.

## 5. Conclusions

The study gives an overview of medicinal plants used by the Maasai of Kenya, disease categories treated with medicinal plants, and their ethnopharmacological uses. Most of the encountered 289 medicinal species were used to treat health disorders in the categories gastrointestinal disorders and respiratory system disorders. This means the two are either the most common health disorders among the Maasai of Kenya or the Maasai community prefers to use traditional medicinal plants to treat disorders in these two categories.

The 289 species encountered in the 19 references were from the Maasai community living in Narok and Kajiado counties in Kenya. No information was available on medicinal plants of the Iljamus and the Laikipia Maasai who inhabit Baringo and Laikipia counties respectively. We also found that medicinal plant knowledge was localized and only shared in part between the counties studied. This implies that people might have developed new knowledge based on the available medicinal plants in their surrounding environments as dictated by ecological and other factors. Based on these findings, it is important to document medicinal plants knowledge in additional villages and counties for knowledge conservation. Therefore, additional research on Maasai medicinal plants from the so far not studied counties is needed to document the ethnomedicinal uses of plants to safeguard their knowledge from extinction. Also, research should be conducted to understand the factors that influence the selection of medicinal plants for treatment.

The Maasai preference for roots compared to other plant parts for medication may be unsustainable and could threaten species availability in the future. To ensure sustainable utilization of plant resources, the community should be encouraged to cultivate medicinal plants. This recommendation would have been meaningless some years ago when the Maasai moved from place to place to feed their cattle, but now that they are becoming more sedentary the suggestion could be relevant. Alternatively, the Maasai could consider the use of other parts of the plant such as the fruits and leaves which may be more sustainable to harvest. In addition, it is necessary to find new ways to harvest and prepare the medicine to protect medicinal plants resources obtained largely from the wild.

Phytochemical and pharmacological analysis should be directed towards the highlighted medicinal species with more than five use reports in each disorder category to understand the rationale for usage. The discovery of more and new medicinal plants could lead to the discovery of new drugs in the future.

## Figures and Tables

**Table 1:** Plant families and their numbers of medicinal plant species used by the Kenya Maasai as cited in 19 references reviewed in this study.

Family	Medicinal Species
Leguminosae	40
Asteraceae	25
Malvaceae	16
Lamiaceae	14
Euphorbiaceae	13
Apocynaceae	8
Rubiaceae	8
Anacardiaceae, Capparaceae, Rutaceae, Vitaceae	7
Oleaceae, Solanaceae	6
Burseraceae, Celastraceae, Meliaceae, Rhamnaceae	5
Acanthaceae, Boraginaceae, Commelinaceae, Primulaceae, Salicaceae	4
Amaranthaceae, Apiaceae, Asparagaceae, Crassulaceae, Ebenaceae, Moraceae, Verbenaceae, Xanthorrhoeaceae	3
Clusiaceae, Araliaceae, Canellaceae, Combretaceae, Cucurbitaceae, Icacinaceae, Olacaceae, Penaeaceae, Plantaginaceae, Poaceae, Podocarpaceae, Polygonaceae, Proteaceae, Ranunculaceae, Rosaceae, Santalaceae, Urticaceae, Zygophyllaceae	2
Linderniaceae, Bignoniaceae, Convolvulaceae, Fagaceae, Hydnoraceae, Hypericaceae, Loganiaceae, Loranthaceae, Musaceae, Myricaceae, Myrtaceae, Opiliaceae, Peraceae, Phyllanthaceae, Piperaceae, Pittosporaceae, Plumbaginaceae, Rhizophoraceae, Salvadoraceae, Sapindaceae, Scrophulariaceae, Talinaceae	1

**Table 2:** The 26 use categories in which the Maasai of Kenya use medicinal plants as encountered in 19 references reviewed in this study. For each use category, the number of species used in the category, the number of use reports encountered for each use category, and the Informant Consensus Factor (IFC) are given.

Categories	Species	Use Reports	ICF Value
Metabolic	1	2	1.00
Social problems	1	2	1.00
Gastrointestinal disorders	171	504	0.66
Respiratory	92	252	0.64
Musculoskeletal disorders	64	134	0.53
Toothache	15	28	0.48
Malaria	78	145	0.47
Urological problems	54	101	0.47
Gynecological disorders	69	127	0.46
Infections	57	93	0.39
Skin disorders	106	158	0.33
Neurological	23	34	0.33
Blood	41	59	0.31
Fever	32	45	0.30
Eye	23	32	0.29
General and unspecified	40	51	0.22
Psychological	14	17	0.19
Venomous animals	20	24	0.17
Nutritional	13	15	0.14
Ear	7	7	0.00
Circulatory	6	6	0.00
Endocrine	4	4	0.00
Injuries	4	4	0.00
Psychological	1	1	0.00
Culture-bound syndromes	1	1	0.00
Immune	1	1	0.00

**Table 3:** Plant species, family, main use, preparation methods, parts used, and use reports for the nine medicinal use categories with more than five use reports per species in the 19 references reviewed in this study.

Medicinal Use Categories	Plant Species	Family	Ethnomedicinal Use (s)	Preparation Method (s)	Part (S) Use	Use-Reports
Gastrointestinal disorder	*Acacia nilotica* (L.) Delile	Leguminosae	Aid digestion, appetite enhancer, diarrhea, dysentery	Decoction	Multiple parts	18
	*Albizia anthelmintica* Brongn.	Leguminosae	Dewormer, diarrhea, emetic, purgative, tapeworm	N/A	Bark, multiple parts	17
	*Warburgia ugandensis* Sprague	Canellaceae	Constipation, stomachache, diarrhea	Decoction	Bark	15
	*Solanum incanum* L.	Solanaceae	Abnominal pain, dyspepsia, indigestion, ringworm, stomachache,	Decoction	Fruit	14
	*Myrsine africana* L.	Primulaceae	Anthelmintic, constipation, purgative, stomachache	N/A	Flower, fruit	11
	*Ximenia americana* L.	Olacaceae	Constipation, stomachache, diarrhea, worms	Decoction	Multiple parts	11
	*Baccharoides lasiopus* (O.Hoffm.) H.Rob.	Compositae	Stomachache, purgative, indigestion	N/A	Multiple parts	10
	*Salvadora persica* L.	Salvadoraceae	Anthelmintic, constipation, stomachache, worms	Decoction	Roots	10
	*Acacia mellifera* (M.Vahl) Benth.	Leguminosae	Appetite enhancer, aid in digestion, Stomachache, reduce vomiting	Decoction	Bark	9
	*Euclea divinorum* Hiern	Ebenaceae	Anthelmintic, constipation, emetics, purgatives, stops vomiting	Decoction	Multiple parts, bark, roots	9
	*Pappea capensis* Eckl. & Zeyh.	Sapindaceae	Diarrhea, facilitate digestion, purgative, stomachache	Decoction	Bark	9
	*Searsia natalensis* (Bernh. ex C.Krauss) F.A.Barkley	Anacardiaceae	stomachache, heartburn, poultice	Decoction	Bark	8
	*Warburgia salutaris* (G.Bertol.) Chiov.	Canellaceae	Bloating, stomachache, poultice	N/A	Bark	8
	*Acacia senegal* (L.) Willd.	Leguminosae	Constipation, diarrhea, stomachache	N/A	Bark, root	7
	*Olea europaea* L.	Oleaceae	Deworming, stomach pain	N/A	Bark	7
	*Acacia tortilis* (Forssk.) Hayne	Leguminosae	Reduce vomiting, indigestion	Pounding	Multiple parts, root	6
	*Aloe volkensii* Engl.	Xanthorrhoeaceae	Diarrhea, stomachache	N/A	Multiple parts	6
	*Ricinus communis* L.	Euphorbiaceae	Purgative, stomachache, diarrhea	Pounding, decoction	Seed, root, multiple parts	6
	*Toddalia asiatica* (L.) Lam.	Rutaceae	stomachache, digestion, emetics	Decoction	Bark	6
	*Turraea mombassana* C. DC.	Meliaceae	Dysentery, emetic	N/A	Root	6
	*Vepris simplicifolia* (Engl.) Mziray	Rutaceae	Stomachache, diarrhea, hepatitis	Decoction	Root, bark, multiple parts	6
	*Boscia angustifolia* A.Rich.	Capparaceae	Induce vomiting, diarrhea, anthelmintic, stomachache	Decoction	Leaf, bark	5
	*Leonotis ocymifolia* var. raineriana (Vis.) Iwarsson	Lamiaceae	Dysentery, indigestion, relieve stomach cramps	N/A	Root, leaf	5
	*Lippia javanica* (Burm.f.) Spreng.	Verbenaceae	Indigestion, tapeworm, Stomachache	Pounding	Leaf	5
	*Prunus africana* (Hook.f.) Kalkman	Rosaceae	Increase appetite, stomachache	Pounding	Leaf	5
	*Tarchonanthus camphoratus* L.	Compositae	Abdominal disorders, diarrhea, tapeworms	Decoction	Leaf, mmultiple parts	5
Respiratory system disorders	*Warburgia ugandensis* Sprague	Canellaceae	Chest complains, colds, coughs, flu, respiratory	N/A	Multiple parts, bark	12
	*Toddalia asiatica* (L.) Lam.	Rutaceae	Bronchial pain, colds, respiratory diseases	N/A	Fruit, root, multiple parts	10
	*Lippia javanica* (Burm.f.) Spreng.	Verbenaceae	Blocked nose bronchitis, colds, coughs, flu, sneezing, stuffy nose	N/A	Multiple parts, leaf, seed	9
	*Tarchonanthus camphoratus* L.	Compositae	Asthma, bronchitis	Decoction	Leaf, Multiple parts,	8
	*Warburgia salutaris* (G.Bertol.) Chiov.	Canellaceae	Chest pain, common cold, coughing, pneumonia, respiratory problems	N/A	Multiple parts, bark	8
	*Acacia nilotica* (L.) Delile	Leguminosae	Chest pains, coughs, pneumonia, tuberculosis, coughs	N/A	Root, bark,	7
	*Acacia mellifera* (M.Vahl) Benth.	Leguminosae	Pneumonia, chest pains, coughs	Decoction	Bark	5
	*Ocimum gratissimum* L.	Lamiaceae	blocked nose, colds	Decoction	Leaf	5
	*Olea europaea* L.	Oleaceae	Colds, influenza, pneumonia, respiratory	N/A	Multiple parts	5
	*Searsia natalensis* (Bernh. ex C.Krauss) F.A.Barkley	Anacardiaceae	chest pain, respiratory problems, influenza, coughs	Pounded	Multiple parts, leaf, root	5
	*Vepris simplicifolia* (Engl.) Mziray	Rutaceae	Pneumonia	N/A	Root, multiple parts	5
	*Salvadora persica* L.	Salvadoraceae	Chest pain, cold, flu	N/A	Roots, bark	5
Musculoskeletal disorders	*Rhamnus prinoides* L’Hér.	Rhamnaceae	Arthritis, backaches, rheumatic	Decoction	Root	11
	*Carissa spinarum* L.	Apocynaceae	Backache, muscle pain, joint problems	Decoction	Root	9
	*Combretum molle* R.Br. ex G.Don	Combretaceae	Backache, pelvic pain	N/A	Root	7
	*Kalanchoe glaucescens* Britten	Crassulaceae	Rheumatic	N/A	Leaf	6
	*Warburgia ugandensis* Sprague	Canellaceae	Muscular pains, weak joints	N/A	Multiple parts, bark	6
	*Acacia nilotica* (L.) Delile	Leguminosae	Arthritis, body aches, painful joints	N/A	N/A	5
	*Zanthoxylum usambarense* (Engl.) Kokwaro	Rutaceae	Rheumatic pain, backache, joints pains	Decoction	Bark, leaf	5
Malaria	*Warburgia ugandensis* Sprague	Canellaceae	Malaria	Decoction	Bark, multiple parts	7
	*Salvadora persica* L.	Salvadoraceae	Malaria	N/A	Root	6
	*Acacia mellifera* (M.Vahl) Benth.	Leguminosae	Malaria	Decoction, pounded	Bark	5
	*Albizia anthelmintica* Brongn.	Leguminosae	Anti-malarial	N/A	Multiple parts, bark	5
Gynaecological disorders	*Acacia drepanolobium* Sjostedt	Leguminosae	Fertility, postpartum pain, cleaning of the uterus after birth	N/A	Root, stem, bark	7
	*Acacia oerfota* (Forssk.) Schweinf.	Leguminosae	Cleaning of the uterus after birth, facilitate lactation, facilitate placenta expulsion after birth	N/A	Root	5
Skin disorder	*Solanum incanum* L.	Solanaceae	Cuts, skin diseases, sores, warts, whitlow	Pounding	Fruit	8
Urological disorder	*Rotheca myricoides* (Hochst.) Steane & Mabb.	Lamiaceae	Gonorrhea, syphilis, STIs	N/A	Roots	7
	*Carissa spinarum* L.	Apocynaceae	Syphilis, gonorrhea	Decoction	Roots	7
Neurological disorders	*Tarchonanthus camphoratus* L.	Compositae	Headache	Burning	N/A	5
Toothache disorders	*Salvadora persica* L.	Salvadoraceae	Dental caries, toothache	Pounding	Stem	6

## Supplementary Materials

The following is available online at https://www.mdpi.com/2223-7747/9/1/44/s1, Supplementary material: A listing of 289 medicinal plants used by the Maasai as documented in 19 references reviewed [9,13,14,16,17,18,19,20,21,22,23,24,25,26,27,28,29,30,31]. The species are sorted by the number of use reports for each of them. The plant families (in parenthesis) are those each species is assigned to in The Plant List.
