# Supplementary material for: Medicinal Plants of the Maasai of Kenya: A Review
Source: Plants (Basel). 2019 Dec 27;9(1):44. doi: 10.3390/plants9010044 (PMC7020225; doi:10.3390/plants9010044)
Supplement: Supplementary file 1 [file plants-09-00044-s001.pdf]

Supplemental material: A listing of 289 medicinal plants used by the Maasai as documented in 19 references reviewed [9,13,14,16−31]. The species are sorted by the number of use reports for each of them. The plant families (in parenthesis) are those each species is assigned to in *The Plant List.*

**Medicinal plants and Families**	**Use reports**
Warburgia ugandensis Sprague (Canellaceae)	57
Acacia nilotica (L.) Delile (Leguminosae)	52
Solanum incanum L. (Solanaceae)	51
Salvadora persica L. (Salvadoraceae)	41
Rhamnus prinoides L'Hér. (Rhamnaceae)	38
Albizia anthelmintica Brongn. (Leguminosae)	34
Carissa spinarum L. (Apocynaceae)	29
Acacia mellifera (M.Vahl) Benth. (Leguminosae)	29
Warburgia salutaris (G.Bertol.) Chiov. (Canellaceae)	25
Vepris simplicifolia (Engl.) Mziray (Rutaceae)	25
Toddalia asiatica (L.) Lam. (Rutaceae)	25
Tarchonanthus camphoratus L. (Asteraceae)	25
Rhamnus staddo A.Rich. (Rhamnaceae)	25
Zanthoxylum usambarense (Engl.) Kokwaro (Rutaceae)	24
Pappea capensis Eckl. & Zeyh. (Sapindaceae)	24
Lippia javanica (Burm.f.) Spreng. (Verbenaceae)	24
Acacia xanthophloea Benth. (Leguminosae)	24
Kigelia africana (Lam.) Benth. (Bignoniaceae)	23
Euclea divinorum Hiern (Ebenaceae)	23
Rotheca myricoides (Hochst.) Steane & Mabb. (Lamiaceae)	22
Olea europaea L. (Oleaceae)	22
Ximenia americana L. (Olacaceae)	21
Searsia natalensis (Bernh. ex C.Krauss) F.A.Barkley (Anacardiaceae)	21
Tamarindus indica L. (Leguminosae)	18
Combretum molle R.Br. ex G.Don (Combretaceae)	18
Myrsine africana L. (Primulaceae)	17
Baccharoides lasiopus (O.Hoffm.) H.Rob. (Asteraceae)	17
Artemisia afra Jacq. ex Willd. (Asteraceae)	16
Acacia tortilis (Forssk.) Hayne (Leguminosae)	15
Acacia drepanolobium Sjostedt (Leguminosae)	15
Euphorbia candelabrum Trémaux ex Kotschy (Euphorbiaceae)	14
Acacia oerfota (Forssk.) Schweinf. (Leguminosae)	14
Grewia damine Gaertn. (Malvaceae)	13
Cordia monoica Roxb. (Boraginaceae)	13
Psiadia punctulata (DC.) Vatke (Asteraceae)	12
Prunus africana (Hook.f.) Kalkman (Rosaceae)	12
Maesa lanceolata Forssk. (Primulaceae)	12

Leonotis nepetifolia (L.) R.Br. (Lamiaceae)	12
Commiphora africana (A.Rich.) Endl. (Burseraceae)	12
Aloe volkensii Engl. (Xanthorrhoeaceae)	12
Achyranthes aspera L. (Amaranthaceae)	12
Ziziphus mucronata Willd. (Rhamnaceae)	11
Sericocomopsis hildebrandtii Schinz (Amaranthaceae)	11
Searsia pyroides (Burch.) Moffett (Anacardiaceae)	11
Maerua triphylla A. Rich. (Capparaceae)	11
Leonotis ocymifolia var. *raineriana* (Vis.) Iwarsson (Lamiaceae)	11
Lantana trifolia L. (Verbenaceae)	11
Grewia villosa Willd. (Malvaceae)	11
Withania somnifera (L.) Dunal (Solanaceae)	10
Schrebera alata (Hochst.) Welw. (Oleaceae)	10
Hibiscus fuscus Garcke (Malvaceae)	10
Gymnosporia heterophylla (Eckl. & Zeyh.) Loes. (Celastraceae)	10
Gardenia ternifolia Schumach. & Thonn. (Rubiaceae)	10
Ficus sur Forssk. (Moraceae)	10
Erythrina abyssinica DC. (Leguminosae)	10
Croton dichogamus Pax (Euphorbiaceae)	10
Boscia angustifolia A.Rich. (Capparaceae)	10
Balanites aegyptiaca (L.) Delile (Zygophyllaceae)	10
Acacia senegal (L.) Willd. (Leguminosae)	10
Acacia gerrardii Benth. (Leguminosae)	10
Acacia brevispica Harms (Leguminosae)	10
Ricinus communis L. (Euphorbiaceae)	9
Osyris lanceolata Hochst. & Steud. (Santalaceae)	9
Ormocarpum trichocarpum (Taub.) Engl. (Leguminosae)	9
Olinia rochetiana A.Juss. (Penaeaceae)	9
Ficus sycomorus L. (Moraceae)	9
Euphorbia tirucalli L. (Euphorbiaceae)	9
Senna didymobotrya (Fresen.) H.S.Irwin & Barneby (Leguminosae)	9
Delonix elata (L.) Gamble (Leguminosae)	9
Clausena anisata (Willd.) Hook.f. ex Benth. (Rutaceae)	9
Aloe secundiflora Engl. (Xanthorrhoeaceae)	9
Turraea mombassana C. DC. (Meliaceae)	8
Ocimum gratissimum L. (Lamiaceae)	8
Microglossa pyrifolia (Lam.) Kuntze (Asteraceae)	8
Catha edulis (Vahl) Endl. (Celastraceae)	8
Acokanthera oppositifolia (Lam.) Codd (Celastraceae)	8
Vernonia brachycalyx O.Hoffm. (Asteraceae)	7
Cordia sinensis Lam. (Boraginaceae)	7
Commelina benghalensis L. (Commelinaceae)	7
Cadaba farinosa Forssk. (Capparaceae)	7
Balanites glabra Mildbr. & Schltr. (Zygophyllaceae)	7

Albizia amara (Roxb.) B.Boivin (Leguminosae)	7
Tetradenia riparia (Hochst.) Codd (Lamiaceae)	6
Steganotaenia araliacea Hochst. (Apiaceae)	6
Scutia myrtina (Burm.f.) Kurz (Rhamnaceae)	6
Sclerocarya birrea subsp. *caffra* (Sond.) Kokwaro (Anacardiaceae)	6
Phragmanthera usuiensis (Oliv.) M.G.Gilbert (Loranthaceae)	6
Kalanchoe glaucescens Britten (Crassulaceae)	6
Hypericum revolutum Vahl (Hypericaceae)	6
Dovyalis abyssinica (A.Rich.) Warb. (Salicaceae)	6
Cynanchum viminale (L.) L. (Apocynaceae)	6
Cissus quadrangularis L. (Vitaceae)	6
Blepharis stuhlmannii Lindau (Acanthaceae)	6
Aneilema aequinoctiale (P.Beauv.) Loudon (Commelinaceae)	6
Adansonia digitata L (Malvaceae)	6
Solanum taitense Vatke (Solanaceae)	5
Solanum aculeastrum Dunal (Solanaceae)	5
Searsia tenuinervis (Engl.) Moffett (Anacardiaceae)	5
Sclerocarya birrea (A.Rich.) Hochst. (Anacardiaceae)	5
Pentas lanceolata (Forssk.) Deflers (Rubiaceae)	5
Olea capensis L. (Oleaceae)	5
Melhania parvifolia Chiov. (Malvaceae)	5
Grewia tephrodermis K. Schum. (Malvaceae)	5
Fuerstia africana T.C.E.Fr. (Lamiaceae)	5
Cussonia holstii Harms ex Engl. (Araliaceae)	5
Commiphora samharensis subsp. *terebinthina* (Vollesen) J.B.Gillett (Burseraceae)	5
Acokanthera schimperi (A.DC.) Schweinf. (Apocynaceae)	5
Acacia seyal Delile (Leguminosae)	5
Acacia kirkii Oliv. (Leguminosae)	5
Acacia hockii De Wild. (Leguminosae)	5
Zanthoxylum chalybeum Engl. (Rutaceae)	4
Turraea robusta Gürke (Meliaceae)	4
Trimeria grandifolia (Hochst.) Warb. (Salicaceae)	4
Strychnos henningsii Gilg (Loganiaceae)	4
Senna italica Mill. (Leguminosae)	4
Sarcostemma stocksii Hook. f. (Apocynaceae)	4
Rhoicissus tridentata (L.f.) Wild & R.B.Drumm. (Vitaceae)	4
Pittosporum viridiflorum Sims (Pittosporaceae)	4
Ormocarpum kirkii S.Moore (Leguminosae)	4
Lippia kituiensis Vatke (Verbenaceae)	4
Hydnora abyssinica A.Br. (Hydnoraceae)	4
Harrisonia abyssinica Oliv. (Rutaceae)	4
Gardenia volkensii K.Schum.(Rubiaceae)	4
Garcinia livingstonei T.Anderson (Clusiaceae)	4
Garcinia dumosa King (Clusiaceae)	4

Ehretia cymosa Thonn. (Boraginaceae)	4
Dombeya burgessiae Gerrard ex Harv. (Malvaceae)	4
Croton megalocarpus Hutch. (Euphorbiaceae)	4
Croton macrostachyus Hochst. ex Delile (Euphorbiaceae)	4
Cotyledon barbeyi Schweinf. ex Baker (Crassulaceae)	4
Commiphora schimperi (O.Bergman) Engl. (Burseraceae)	4
Boscia coriacea Graells (Capparaceae)	4
Aspilia pluriseta Schweinf. ex Schweinf. (Asteraceae)	4
Acacia reficiens subsp. *misera* (Vatke) Brenan (Leguminosae)	4
Acacia ancistroclada Brenan (Leguminosae)	4
Urtica massaica Mildbr. (Urticaceae)	3
Talinum portulacifolium (Forssk.) Asch. ex Schweinf. (Talinaceae)	3
Sphaeranthus confertifolius Robyns (Asteraceae)	3
Solanecio mannii (Hook.f.) C.Jeffrey (Asteraceae)	3
Sida tenuicarpa Vollesen (Malvaceae)	3
Senna didymobotrya (Fresen.) H.S.Irwin & Barneby (Leguminosae)	3
Rapanea melanophloeos (L.) Mez (Primulaceae)	3
Phyllanthus urinaria L. (Phyllanthaceae)	3
Ozoroa insignis Delile (Anacardiaceae)	3
Opilia campestris Engl. (Opiliaceae)	3
Olea europaea subsp. *cuspidata* (Wall. & G.Don) Cif. (Oleaceae)	3
Maerua angolensis DC. (Capparaceae)	3
Jasminum fluminense Vell. (Oleaceae)	3
Heteromorpha trifoliata (H.L.Wendl.) Eckl. & Zeyh. (Apiaceae)	3
Helichrysum forskahlii (J.F.Gmel.) Hilliard & B.L.Burtt (Asteraceae)	3
Ficus thonningii Blume (Moraceae)	3
Euphorbia meridionalis P.R.O.Bally & S.Carter (Euphorbiaceae)	3
Euphorbia gossypina Pax (Euphorbiaceae)	3
Euphorbia cuneata Vahl (Euphorbiaceae)	3
Dombeya rotundifolia (Hochst.) Planch. (Malvaceae)	3
Cyphostemma cyphopetalum var. *nodiglandulosum* (T.C.E.Fr.) Verdc. (Vitaceae)	3
Croton megalocarpoides Friis & M.G.Gilbert (Euphorbiaceae)	3
Commiphora campestris Engl. (Burseraceae)	3
Cissus rotundifolia Vahl (Vitaceae)	3
Cassine buchananii Loes. (Celastraceae)	3
Albizia lebbeck (L.) Benth. (Leguminosae)	3
Aerva lanata (L.) Juss. (Amaranthaceae)	3
Acacia etbaica Schweinf. (Leguminosae)	3
Acacia elatior Brenan (Leguminosae)	3
Vernonia abbotiana O.Hoffm. (Asteraceae)	2
Urtica dioica L. (Urticaceae)	2
Turraea abyssinica Hochst. (Meliaceae)	2
Trimeria bakeri Gilg (Salicaceae)	2
Tinnea aethiopica Kotschy ex Hook.f. (Lamiaceae)	2

Tetradenia multiflora (Benth.) Phillipson (Lamiaceae)	2
Syzygium cordatum Hochst. ex Krauss (Myrtaceae)	2
Sporobolus stapfianus Gand. (Poaceae)	2
Solanum mauense Bitter (Solanaceae)	2
Solanecio angulatus (Vahl) C.Jeffrey (Asteraceae)	2
Senna septemtrionalis (Viv.) H.S.Irwin & Barneby (Leguminosae)	2
Searsia natalensis (Bernh. ex C.Krauss) F.A.Barkley (Anacardiaceae)	2
Rumex usambarensis (Dammer) Dammer (Polygonaceae)	2
Pyrenacantha malvifolia Engl. (Icacinaceae)	2
Podocarpus latifolius (Thunb.) R.Br. ex Mirb. (Podocarpaceae)	2
Plantago major L. (Plantaginaceae)	2
Piper capense L.f. (Piperaceae)	2
Pavonia urens Cav. (Malvaceae)	2
Pavonia burchellii (DC.) R.A.Dyer (Malvaceae)	2
Morella salicifolia var. *kilimandscharica* (Engl.) Verdc. & Polhill (Myricaceae)	2
Mondia whitei (Hook.f.) Skeels (Apocynaceae)	2
Lycium europaeum L. (Solanaceae)	2
Leucas tomentosa Gürke (Lamiaceae)	2
Leucas jamesii Baker (Lamiaceae)	2
Justicia odora (Forssk.) Lam. (Acanthaceae)	2
Ipomoea jaegeri Pilg. (Convolvulaceae)	2
Indigofera erecta Thunb. (Leguminosae)	2
Gutenbergia cordifolia Benth. ex Oliv. (Asteraceae)	2
Galium aparinoides Forssk. (Rubiaceae)	2
Faurea saligna Harv. (Proteaceae)	2
Embelia schimperi Vatke (Primulaceae)	2
Dombeya torrida (J.F.Gmel.) Bamps (Malvaceae)	2
Cucumis ficifolius A.Rich. (Cucurbitaceae)	2
Croton dichogamus Pax (Euphorbiaceae)	2
Croton zambalensis Merr. (Euphorbiaceae)	2
Craterostigma plantagineum Hochst. (Linderniaceae)	2
Commelina africana L. (Commelinaceae)	2
Chloris virgata Sw. (Poaceae)	2
Cassine aethiopica Thunb. (Celastraceae)	2
Calodendrum capense (L.f.) Thunb. (Rutaceae)	2
Buddleja polystachya Fresen. (Scrophulariaceae)	2
Blepharis linariifolia Pers. (Acanthaceae)	2
Bauhinia thonningii Schum. (Leguminosae)	2
Azadirachta indica A. Juss. (Meliaceae)	2
Aspilia mossambicensis (Oliv.) Wild (Asteraceae)	2
Albizia berteriana (DC.) Fawc. & Rendle (Leguminosae)	2
Acacia robusta Burch. (Leguminosae)	2
Acacia lahai Benth. (Leguminosae)	2
Abutilon longicuspe Hochst. ex A.Rich. (Malvaceae)	2
Zehneria scabra Sond. (Cucurbitaceae)	1

Ximenia caffra Sond. (Olacaceae)	1
Viscum tuberculatum A.Rich. (Santalaceae)	1
Vernonia brachycalyx O.Hoffm (Asteraceae)	1
Vernonia auriculifera Hiern (Asteraceae)	1
Vangueria madagascariensis J.F.Gmel. (Rubiaceae)	1
Vangueria infausta Burch. (Rubiaceae)	1
Tripteris vaillantii Decne. (Asteraceae)	1
Thunbergia fischeri Engl. (Acanthaceae)	1
Terminalia brownii Fresen. (Combretaceae)	1
Sphaeranthus suaveolens (Forssk.) DC. (Asteraceae)	1
Schinus molle L. (Anacardiaceae)	1
Sansevieria volkensii Gürke (Asparagaceae)	1
Sansevieria suffruticosa N.E.Br. (Asparagaceae)	1
Rubus steudneri Schweinf. (Rosaceae)	1
Rhynchosia calycosa Hemsl. (Leguminosae)	1
Rhoicissus erythrodes (Fresen.) Planch. (Vitaceae)	1
Ranunculus oreophytus Delile (Ranunculaceae)	1
Quercus rotundifolia Lam. (Fagaceae)	1
Pterolobium stellatum (Forssk.) Brenan (Leguminosae)	1
Protea gaguedi J.F.Gmel. (Proteaceae)	1
Plumbago zeylanica L. (Plumbaginaceae)	1
Plantago palmata Hook.f. (Plantaginaceae)	1
Persicaria setosula (A.Rich.) K.L.Wilson (Polygonaceae)	1
Olinia usambarensis Gilg ex Engl. (Penaeaceae)	1
Oldenlandia monanthos (Hochst. ex A.Rich.) Hiern (Rubiaceae)	1
Musa acuminata Colla (Musaceae)	1
Leucas calostachys Oliv. (Lamiaceae)	1
Leonotis ocymifolia (Burm.f.) Iwarsson (Lamiaceae)	1
Laphangium luteoalbum (L.) Tzvelev (Asteraceae)	1
Kalanchoe densiflora Rolfe (Crassulaceae)	1
Jasminum abyssinicum Hochst. ex DC. (Oleaceae)	1
Indigofera arrecta A.Rich. (Leguminosae)	1
Hymenodictyon parvifolium Oliv. (Rubiaceae)	1
Hoslundia opposita Vahl (Lamiaceae)	1
Hirpicium diffusum (O.Hoffm.) Roessler (Asteraceae)	1
Helinus mystacinus (Aiton) E.Mey. ex Steud. (Rhamnaceae)	1
Gymnosporia senegalensis (Lam.) Loes. (Celastraceae)	1
Gutenbergia rueppellii Sch.Bip. (Asteraceae)	1
Grewia similis K.Schum. (Malvaceae)	1
Flacourtia indica (Burm.f.) Merr. (Salicaceae)	1
Euryops jacksonii S.Moore (Asteraceae)	1
Euphorbia uhligiana Pax (Euphorbiaceae)	1
Euphorbia robecchii Pax (Euphorbiaceae)	1
Euclea racemosa L. (Ebenaceae)	1

Ekebergia capensis Sparrm. (Meliaceae)	1
Echinops sphaerocephalus L. (Asteraceae)	1
Dregea schimperi (Decne.) Bullock (Apocynaceae)	1
Dombeya kirkii Mast. (Malvaceae)	1
Diospyros abyssinica (Hiern) F.White (Ebenaceae)	1
Cyphostemma serpens (Hochst. ex A.Rich.) Desc. (Vitaceae)	1
Cyphostemma cyphopetalum (Fresen.) Desc. ex Wild & R.B.Drumm. (Vitaceae)	1
Cynanchum altiscandens K.Schum. (Apocynaceae)	1
Cussonia spicata Thunb. (Araliaceae)	1
Crotalaria rotundifolia J.F.Gmel. (Leguminosae)	1
Crotalaria agatiflora Schweinf. (Leguminosae)	1
Crepis carbonaria Sch.Bip. (Asteraceae)	1
Cordia africana Lam. (Boraginaceae)	1
Commiphora swynnertonii Burtt (Burseraceae)	1
Clutia abyssinica Jaub. & Spach (Peraceae)	1
Clerodendrum rotundifolium Oliv. (Lamiaceae)	1
Clematis brachiata Thunb. (Ranunculaceae)	1
Centrapalus pauciflorus (Willd.) H.Rob. (Asteraceae)	1
Cassipourea malosana (Baker) Alston (Rhizophoraceae)	1
Capparis tomentosa Lam. (Capparaceae)	1
Capparis fascicularis DC. (Capparaceae)	1
Cajanus cajan (L.) Millsp. (Leguminosae)	1
Baccharoides adoensis (Sch.Bip. ex Walp.) H.Rob. (Asteraceae)	1
Asparagus africanus Lam. (Asparagaceae)	1
Apodytes dimidiata E.Mey. ex Arn. (Icacinaceae)	1
Anthriscus sylvestris (L.) Hoffm. (Apiaceae)	1
Aneilema spekei C.B.Clarke (Commelinaceae)	1
Aloe vera (L.) Burm.f. (Xanthorrhoeaceae)	1
Afrocarpus falcatus (Thunb.) C.N.Page (Podocarpaceae)	1
Acacia sieberiana DC. (Leguminosae)	1
Acacia ericifolia Benth. (Leguminosae)	1
